# Recalibration of a Deep Learning Model for Low-Dose Computed Tomographic Images to Inform Lung Cancer Screening Intervals

**DOI:** 10.1001/jamanetworkopen.2023.3273

**Published:** 2023-03-16

**Authors:** Rebecca Landy, Vivian L. Wang, David R. Baldwin, Paul F. Pinsky, Li C. Cheung, Philip E. Castle, Martin Skarzynski, Hilary A. Robbins, Hormuzd A. Katki

**Affiliations:** 1 Division of Cancer Epidemiology and Genetics, National Cancer Institute, National Institutes of Health, Department of Health and Human Services, Bethesda, Maryland; 2 School of Medicine, Nottingham University Hospitals and the University of Nottingham, Nottingham, United Kingdom; 3 Division of Cancer Prevention, National Cancer Institute, National Institutes of Health, Department of Health and Human Services, Bethesda, Maryland; 4 Genomic Epidemiology Group, International Agency for Research on Cancer, Lyon, France

## Abstract

**Importance:**

Annual low-dose computed tomographic (LDCT) screening reduces lung cancer mortality, but harms could be reduced and cost-effectiveness improved by reusing the LDCT image in conjunction with deep learning or statistical models to identify low-risk individuals for biennial screening.

**Objective:**

To identify low-risk individuals in the National Lung Screening Trial (NLST) and estimate, had they been assigned a biennial screening, how many lung cancers would have been delayed 1 year in diagnosis.

**Design, Setting, and Participants:**

This diagnostic study included participants with a presumed nonmalignant lung nodule in the NLST between January 1, 2002, and December 31, 2004, with follow-up completed on December 31, 2009. Data were analyzed for this study from September 11, 2019, to March 15, 2022.

**Exposures:**

An externally validated deep learning algorithm that predicts malignancy in current lung nodules using LDCT images (Lung Cancer Prediction Convolutional Neural Network [LCP-CNN]; Optellum Ltd) was recalibrated to predict 1-year lung cancer detection by LDCT for presumed nonmalignant nodules. Individuals with presumed nonmalignant lung nodules were hypothetically assigned annual vs biennial screening based on the recalibrated LCP-CNN model, Lung Cancer Risk Assessment Tool (LCRAT + CT [a statistical model combining individual risk factors and LDCT image features]), and the American College of Radiology recommendations for lung nodules, version 1.1 (Lung-RADS).

**Main Outcomes and Measures:**

Primary outcomes included model prediction performance, the absolute risk of a 1-year delay in cancer diagnosis, and the proportion of people without lung cancer assigned a biennial screening interval vs the proportion of cancer diagnoses delayed.

**Results:**

The study included 10 831 LDCT images from patients with presumed nonmalignant lung nodules (58.7% men; mean [SD] age, 61.9 [5.0] years), of whom 195 were diagnosed with lung cancer from the subsequent screen. The recalibrated LCP-CNN had substantially higher area under the curve (0.87) than LCRAT + CT (0.79) or Lung-RADS (0.69) to predict 1-year lung cancer risk (*P* < .001). If 66% of screens with nodules were assigned to biennial screening, the absolute risk of a 1-year delay in cancer diagnosis would have been lower for recalibrated LCP-CNN (0.28%) than LCRAT + CT (0.60%; *P* = .001) or Lung-RADS (0.97%; *P* < .001). To delay only 10% of cancer diagnoses at 1 year, more people would have been safely assigned biennial screening under LCP-CNN than LCRAT + CT (66.4% vs 40.3%; *P* < .001).

**Conclusions and Relevance:**

In this diagnostic study evaluating models of lung cancer risk, a recalibrated deep learning algorithm was most predictive of 1-year lung cancer risk and had least risk of 1-year delay in cancer diagnosis among people assigned biennial screening. Deep learning algorithms could prioritize people for workup of suspicious nodules and decrease screening intensity for people with low-risk nodules, which may be vital for implementation in health care systems.

## Key Points

**Question:**

Can a deep learning algorithm reuse a lung screening low-dose computed tomographic image to safely assign individuals with nodules to 1- vs 2-year screening?

**Findings:**

Using data from 10 831 low-dose computed tomographic images with lung nodules from patients in the National Lung Screening Trial, this diagnostic study showed that a recalibrated deep learning algorithm was able to predict lung cancer diagnosis at a 1-year screen with good discrimination. The deep learning algorithm outperformed current American College of Radiology guidelines and statistical models.

**Meaning:**

These findings suggest that deep learning algorithms could be used to assign individuals to 2-year screening intervals, reducing the harms of screening and potentially making screening cost-effective in some health care systems.

## Teaser

This diagnostic study uses deep learning algorithms to evaluate 4 models that use computed tomographic imaging features of a presumed nonmalignant lung nodule to predict lung cancer detection at 1 year among patients in the National Lung Screening Trial.

## Introduction

Two trials, the National Lung Screening Trial (NLST)^1^ and NELSON,^2^ demonstrated that regular low-dose computed tomographic (LDCT) screening of high-risk individuals who ever smoked reduces lung cancer mortality. However, the chance of finding a lung nodule without a verified cancer by the 1-year follow up (abnormal presumed nonmalignant screen result) can be high with annual screening; 23% of NLST screens met this criteria.^1^ Biennial screening may be safe for sufficiently low-risk individuals while improving efficiency, reducing costs, and lessening harms from screening.^2,3^ People whose CT images show no or only very small nodules are classified as having negative findings and are the best candidates for biennial screening, as recommended by the NHS (National Health Service) England Targeted Lung Health Checks Programme.^4^

Biennial screening may also be appropriate for some people with presumed nonmalignant lung nodules who have a higher risk of future lung cancer compared with those with negative screen results.^5^ Importantly, screening intervals of more than 1 year may make screening cost-effective and/or feasible for countries with more limited health care resources. Previous researchers (including D.R.B., L.C.C., H.A.R., and H.A.K.)^6,7^ developed the Lung Cancer Risk Assessment Tool (LCRAT) + CT, a statistical model combining individual risk factors with CT image features (for CT images with no nodules) or lung nodule features (for CT images with nodules) to predict the risk of 1-year lung cancer detection on CT and identify which individuals might safely be assigned to biennial screening.

The American College of Radiology management recommendations for individuals with lung nodules (Lung-RADS), version 1.1, assigns nodules to 5 categories (2, 3, 4A, 4B, and 4X).^8^ The longest screening interval assigned is 1 year for individuals with a score of 2, estimated to apply to 90% of individuals with nodules, including those with a prior solid nodule less than 6 mm in diameter or a new solid nodule less than 4 mm.

Recently, deep learning algorithms applied to CT images of lung nodules have demonstrated substantial improvement in the accuracy of immediate malignant classification of lung nodules vs statistical models.^9,10,11,12,13^ These supervised deep learning algorithms are trained to distinguish CT screens that show lung cancers from those containing nodules but not lung cancer. However, to our knowledge, there is no proposed use of deep learning algorithms on images to predict risk of future disease (excluding cases diagnosed before the next screen) and thereby personalize the length of screening intervals. Herein we demonstrate a proof of principle by evaluating 4 methods that use CT imaging features of a presumed nonmalignant lung nodule to predict lung cancer detection on CT in 1 year: (1) Lung Cancer Prediction Convolutional Neural Network (LCP-CNN; a deep learning algorithm developed by Optellum Ltd), (2) LCRAT + CT, (3) LCRAT plus LCP-CNN, and (4) Lung-RADS. We assigned individuals with presumed nonmalignant lung nodules to annual vs biennial screening based on their lung cancer risk and report the absolute risk of delaying a cancer diagnosis, as well as the tradeoff of the proportion of people without lung cancer assigned biennial screening vs the proportion of cancer diagnoses delayed.

## Methods

The NLST randomized 53 452 individuals aged 55 to 74 years between January 1, 2002, and December 31, 2004, to 3 annual screens with LDCT (n = 26 722) or single-view posteroanterior chest radiography (n = 26 730).^14^ Institutional review boards at each center approved the study, and each person provided written consent to participate in the study. We followed the Standards for Reporting of Diagnostic Accuracy (STARD) reporting guideline for diagnostic studies.

### Study Procedure

We used data from the baseline (T0) and 1-year (T1) NLST LDCT screens to examine the risk of a lung cancer diagnosis resulting from the CT screen obtained 1 year after an abnormal T0 or T1 CT screen result that had at least 1 solid nodule with maximum diameter 5 mm or larger (according to the Optellum Ltd algorithm) without a cancer diagnosis before the next screen. The longest nodule diameter reported in the NLST metadata was 4 mm for 15% of the selected screens, though an algorithm developed by Optellum Ltd recorded the maximum diameter as at least 5 mm for all screens used in this study. Screens with only nonsolid or part-solid noncalcified nodules were excluded. A CONSORT flowchart is provided in eFigure 1 in Supplement 1. Follow-up for cancer incidence was available until December 31, 2009.

We considered a cancer to be diagnosed as the result of a CT screen using the linked-year method as described previously.^15^ A cancer was considered linked to the screen if the screen result was abnormal and there was a chain of diagnostic procedures leading from the screen to diagnosis without more than 1 year between events, where an abnormal screen result was defined as a screen with a noncalcified nodule 4 mm or larger in any diameter or an abnormality such as adenopathy or effusion. We used cancers diagnosed under the linked-year method as a result of the following screen (ie, the screen after the abnormal screen result on which the algorithm or model estimate was based) to define 1-year lung cancer risk.

The LCP-CNN is an externally validated deep learning algorithm developed to distinguish malignant and benign lung nodules^9,11^ (eMethods in Supplement 1). The LCP-CNN score has only been validated for solid nodules with maximum diameter of at least 5 mm; therefore, only these nodules are included in this analysis. Our analysis was performed at the level of individual screens; when multiple nodules were found on a CT image (38% of images), the highest LCP-CNN score (representing immediate lung cancer risk) across nodules was assigned to the screen.

We used CT image data to predict 1-year lung cancer risk and compare the performance of LCP-CNN, LCRAT + CT, LCRAT plus LCP-CNN, and Lung-RADS. First, because LCP-CNN predicts risk of immediate malignancy for a lung nodule, we used logistic regression to recalibrate the LCP-CNN score to predict 1-year screen-detected lung cancer risk (eMethods in Supplement 1). Because LCP-CNN was partially trained on NLST data, for each screen we used an 8-fold cross-validated LCP-CNN score that was not trained on that screen. Second, the LCRAT + CT is a previously developed, individualized risk model combining prescreening risk factors via the LCRAT^16^ with image features of a negative CT screen result^6^ or nodule features^17^ to estimate 1-year screen detection risk. The LCRAT + CT model was also trained on NLST data; more details on LCRAT and LCRAT + CT are available in eMethods in Supplement 1. Third, to assess whether there was increased risk stratification by combining LCP-CNN with prescreening risk, we developed a logistic regression model for 1-year screen-detected cancer risk using 1-year LCRAT risk and LCP-CNN score (LCRAT plus LCP-CNN). Fourth, we considered Lung-RADS, version 1.1.^8^

### Statistical Analysis

Data were analyzed from from September 11, 2019, to March 15, 2022. Model validity was assessed using calibration (ratio of expected to observed lung cancer cases) and discrimination (optimism-adjusted area under the curve [AUC]^18^) for LCRAT + CT, LCP-CNN, and LCRAT plus LCP-CNN. We used each of these methods to assign annual vs biennial screening (for individuals with risks above and below a threshold, respectively) following an abnormal result of a presumed nonmalignant CT screen result (where a lung nodule was identified without a verified cancer by 1-year follow up) in the NLST. We calculated the absolute risk of delaying a cancer diagnosis by 1 year among the individuals assigned to biennial screening when 66%, 80%, and 90% of individuals at lowest risk were assigned biennial screening, and the proportion of individuals at lowest risk assigned to biennial screening when 5%, 10%, 20%, and 35% of cases were delayed 1 year in diagnosis. We consider a case to be delayed 1 year in diagnosis if they were assigned biennial screening and their cancer was detected on the subsequent year’s screen. We report overall results and those stratified by nodule size; for results stratified by nodule size, the threshold used is the overall threshold, not a threshold specific to nodules that size.

Sensitivity analyses were performed using 1 screen per person, selected at random. In addition, since individuals with a Lung-RADS score greater than 2 are currently recommended to be followed up sooner than 1 year, we restricted these analyses to individuals with a Lung-RADS score of 2, the individuals with a nodule who would be assigned an annual screening interval under the current standard of care. Data were analyzed using R, version 4.2.1 (R Program for Statistical Computing). Two-sided *P* < .05 indicated statistical significance.

## Results

There were 10 831 abnormal presumed nonmalignant screen results with a nodule measuring 5 mm or more by the Optellum Ltd algorithm (5359 T0 and 5472 T1) (58.7% from men and 41.3% from women; mean [SD] age, 61.9 [5.0] years) from 7495 individuals (eMethods in Supplement 1), and 195 screen-detected lung cancers. Table 1 shows characteristics of the patients with nodules included in this study. The 1-year lung cancer risk among those with a presumed nonmalignant abnormal screen result was 3.6-fold greater than the mean prescreening risk for all screens included in this study, as calculated by LCRAT (1.8% vs 0.5%; *P* < .001). The 3 models (LCP-CNN, LCRAT + CT, and LCRAT plus LCP-CNN) had good cross-validated internal calibration (all *P* > .77) (Table 2 and eMethods in Supplement 1). One hundred six cancers in this study (54.4%) were diagnosed at stage 1A.

**Table 1.** Patient Characteristics for Screens Included in the Study

Characteristic	Patient group, No. (%)		
	Cancer (n = 195)	Benign (n = 10 636)	Total (N = 10 831)
Sex			
Men	102 (52.3)	6258 (58.8)	6360 (58.7)
Women	93 (47.7)	4378 (41.2)	4471 (41.3)
Largest nodule diameter (clinician-stated, from NLST metadata), mm			
NA^a^	1 (0.5)	32 (0.3)	33 (0.3)
4-5^b^	22 (11.3)	3808 (35.8)	3830 (35.4)
6-7	39 (20.0)	3290 (30.9)	3329 (30.7)
8-10	47 (24.1)	1976 (18.6)	2023 (18.7)
11-13	41 (21.0)	664 (6.2)	705 (6.5)
≥14	45 (23.1)	866 (8.1)	911 (8.4)
Lung-RADS score^c^			
2	69 (35.4)	7026 (66.1)	7095 (65.5)
3	19 (9.7)	1613 (15.2)	1632 (15.1)
3 or 4A	2 (1.0)	83 (0.8)	85 (0.8)
3, 4A, or 4B	11 (5.6)	141 (1.3)	152 (1.4)
4A	48 (24.6)	1194 (11.2)	1242 (11.5)
4A or 4B	11 (5.6)	105 (1.0)	116 (1.1)
4B	35 (17.9)	474 (4.5)	509 (4.7)
Age, y			
55-59	42 (21.5)	3726 (35.0)	3768 (34.8)
60-64	67 (34.4)	3392 (31.9)	3459 (31.9)
65-69	61 (31.3)	2234 (21.0)	2295 (21.2)
70-75	25 (12.8)	1284 (12.1)	1309 (12.1)
No. of nodules^d^			
1	103 (52.8)	6594 (62.0)	6697 (61.8)
2	48 (24.6)	2285 (21.5)	2333 (21.5)
3-5	40 (20.5)	1561 (14.7)	1601 (14.8)
≥6	4 (2.1)	196 (1.8)	200 (1.8)
Nodule location^e^			
Right middle lobe	27 (13.8)	2259 (21.2)	2286 (21.1)
Upper lobe	142 (72.8)	5610 (52.7)	5752 (53.1)
Lower lobe	91 (46.7)	5792 (54.5)	5883 (54.3)
Nodule spiculation			
Nonspiculated	129 (66.2)	9475 (89.1)	9604 (88.7)
Spiculated	66 (33.8)	1161 (10.9)	1227 (11.3)
Time since quit smoking, y			
0	113 (57.9)	5553 (52.2)	5666 (52.3)
1-4	25 (12.8)	1291 (12.1)	1316 (12.2)
5-9	28 (14.4)	1597 (15.0)	1625 (15.0)
10-14	25 (12.8)	1737 (16.3)	1762 (16.3)
15-19	4 (2.1)	447 (4.2)	451 (4.2)
≥20	0	11 (0.1)	11 (0.1)
No. of smoking pack-years			
30-39	24 (12.3)	2470 (23.2)	2494 (23.0)
40-49	37 (19.0)	2898 (27.2)	2935 (27.1)
≥50	134 (68.7)	5268 (49.5)	5402 (49.9)

Abbreviations: Lung-RADS, American College of Radiology recommendations for lung nodules, version 1.1; NA, not available; NLST, National Lung Screening Trial.

^a^ Screen result was positive for a reason (ie, suspicious abnormalities) other than a nodule 4 mm in diameter or larger. These screens are recorded as having 1 nodule.

^b^ Nodule size is reported according to the NLST metadata, though all screens included in the study had at least 1 nodule 5 mm in diameter or larger according to the Optellum Ltd algorithm.

^c^ Insufficient information was available to assign some individuals to 1 Lung-RADS category, so they were assigned to a group category (eg, 3 or 4A).

^d^ As reported in NLST metadata.

^e^ Screens with more than 1 nodule can contain nodules in more than 1 location. Thus, nodules from each location sum to a total greater than the number of screens with at least 1 nodule.

**Table 2.** Model Performance and the Absolute Risk of Delaying a Cancer Diagnosis, for a Range of Risk Thresholds, for Each Model

Model	No. of screens	Risk under the specified model, median (IQR), %	AUC^a^	No. of cancers		Calibration, ratio of expected to observed cases (95% CI)	Absolute risk of delaying a cancer in diagnosis, No. of cancers delayed in diagnosis/No. of individuals assigned biennial screening (%)		
				Expected	Observed		66%	80%	90%
**All nodules** ^b^									
LCRAT + CT	10 831	1.0 (0.5-2.2)	0.79	204	195	1.05 (0.91-1.21)	43/7148 (0.60)	71/8665 (0.82)	110/9748 (1.13)
LCP-CNN	10 831	0.5 (0.2-1.7)	0.87	196	195	1.00 (0.87-1.15)	20/7148 (0.28)	38/8665 (0.44)	72/9748 (0.74)
LCRAT plus LCP-CNN	10 831	0.5 (0.2-1.6)	0.87	196	195	1.00 (0.87-1.15)	18/7148 (0.25)	37/8665 (0.43)	72/9748 (0.74)
**Nodules 4-5 mm in diameter**									
LCRAT + CT	3830	0.5 (0.3-0.9)	0.68	26	22	1.20 (0.79-1.82)	16/3515 (0.46)	20/3758 (0.53)	20/3817 (0.52)
LCP-CNN	3830	0.3 (0.1-0.6)	0.73	24	22	1.09 (0.72-1.66)	8/3260 (0.25)	12/3619 (0.33)	17/3779 (0.45)
LCRAT plus LCP-CNN	3830	0.2 (0.1-0.6)	0.75	23	22	1.06 (0.70-1.61)	8/3270 (0.24)	12/3617 (0.33)	17/3779 (0.45)
**Nodules 6-7 mm in diameter**									
LCRAT + CT	3329	0.8 (0.5-1.5)	0.73	39	39	0.99 (0.72-1.35)	18/2592 (0.69)	28/3065 (0.91)	35/3255 (1.08)
LCP-CNN	3329	0.4 (0.1-1.2)	0.83	37	39	0.96 (0.70-1.31)	7/2381 (0.29)	16/2890 (0.55)	25/3171 (0.79)
LCRAT plus LCP-CNN	3329	0.4 (0.1-1.2)	0.83	37	39	0.96 (0.70-1.31)	6/2377 (0.25)	16/2882 (0.56)	23/3164 (0.73)
**Nodules 8-10 mm in diameter**									
LCRAT + CT	2023	2.0 (1.1-3.4)	0.74	54	47	1.15 (0.86-1.53)	6/817 (0.73)	13/1290 (1.01)	27/1725 (1.57)
LCP-CNN	2023	0.9 (0.3-2.6)	0.86	44	47	0.93 (0.70-1.24)	3/1068 (0.28)	6/1439 (0.42)	15/1758 (0.85)
LCRAT plus LCP-CNN	2023	0.9 (0.3-2.6)	0.87	44	47	0.93 (0.70-1.24)	2/1062 (0.19)	5/1434 (0.35)	17/1758 (0.97)
**Nodules 11-13 mm in diameter**									
LCRAT + CT	705	4.3 (2.5-7.2)	0.72	39	41	0.95 (0.70-1.29)	0/73	4/198 (2.02)	7/363 (1.93)
LCP-CNN	705	2.3 (0.8-5.6)	0.83	31	41	0.77 (0.57-1.04)	0/201	2/346 (0.58)	9/483 (1.86)
LCRAT plus LCP-CNN	705	2.1 (0.8-5.5)	0.83	32	41	0.79 (0.58-1.07)	0/211	2/361 (0.55)	8/486 (1.65)
**Nodules ≥14 mm in diameter**									
LCRAT + CT	911	3.5 (2.1-6.0)	0.65	45	45	1.00 (0.75-1.34)	3/141 (2.13)	7/333 (2.10)	21/563 (3.73)
LCP-CNN	911	3.3 (1.1-7.9)	0.83	58	45	1.28 (0.96-1.71)	2/226 (0.88)	2/353 (0.57)	6/531 (1.13)
LCRAT plus LCP-CNN	911	3.5 (1.1-7.7)	0.83	58	45	1.28 (0.96-1.72)	2/214 (0.93)	2/353 (0.57)	2/536 (1.37)

Abbreviations: AUC, area under the curve; LCP-CNN, Lung Cancer Prediction Convolutional Neural Network; LCRAT + CT, Lung Cancer Risk Assessment Tool plus computed tomography.

^a^ Optimism corrected for LCP-CNN and LCRAT plus LCP-CNN, to reflect that LCP-CNN was refit to these data.

^b^ A total of 33 screens had positive results for a reason (ie, suspicious abnormalities) other than a nodule 4 mm in diameter or larger.

The AUC for the recalibrated LCP-CNN score (0.87) was greater than that of LCRAT + CT (0.79; *P* < .001) and Lung-RADS (0.69; *P* < .001) and did not differ from the combined LCRAT plus LCP-CNN (0.87; *P* = .12). A sensitivity analysis refitting the LCRAT + CT model to this data set (excluding whether there was any suspicious change in attenuation, so the model would converge in this restricted sample) showed similar results to the original LCRAT + CT model (AUC, 0.77). When stratified by nodule size, the AUC ranged from 0.65 to 0.74 for LCRAT + CT, and 0.73 to 0.86 for LCP-CNN (Table 2). The recalibrated LCP-CNN and LCRAT plus LCP-CNN identified more people either at very low risk or very high risk than LCRAT + CT (eFigure 2 in Supplement 1). When the analyses were restricted to 1 screen per person, the AUCs for each of the 4 methods were similar to those in the main analyses (eMethods in Supplement 1).

### Tradeoff Between Assigning People to Biennial Screening vs Cancers Delayed 1 Year in Diagnosis

The Figure illustrates the tradeoff between assigning people with abnormal presumed nonmalignant screen results in the NLST (which imposed yearly screening for everyone) who were not diagnosed with lung cancer before the next screen for biennial screening vs a potential 1-year delay in lung cancer diagnosis. Because Lung-RADS would assign 66% of screens with abnormal presumed nonmalignant results to biennial screening at a threshold of 2 or less, we set thresholds for LCRAT + CT, LCP-CNN, and LCRAT plus LCP-CNN that also refer 66% for biennial screening (at thresholds of 1.58%, 1.04%, and 1.02%, respectively). If everyone was assigned biennial screening, the absolute risk of delaying a cancer diagnosis was 1.80%. In contrast, the absolute risk of delaying a cancer diagnosis was 0.97% under Lung-RADS, 0.60% under LCRAT + CT (*P* = .01 vs Lung-RADS), and 0.28% under recalibrated LCP-CNN (*P* < .001 vs Lung-RADS and *P* = .005 vs LCRAT + CT) (Table 2). Using the same thresholds, LCP-CNN had a lower risk of delaying a cancer diagnosis than LCRAT + CT across all nodule sizes. Table 2 also shows results where 80% and 90% of screens are assigned biennial screening. When 1 screen per person was used in the analysis, Lung-RADS would assign 64% of screens with abnormal presumed nonmalignant results to biennial screening, and the absolute risks of delaying a lung cancer diagnosis under LCRAT + CT, LCP-CNN, and Lung-RADS were similar to those in the main analyses (eTable 4 in Supplement 1).

**Figure.**
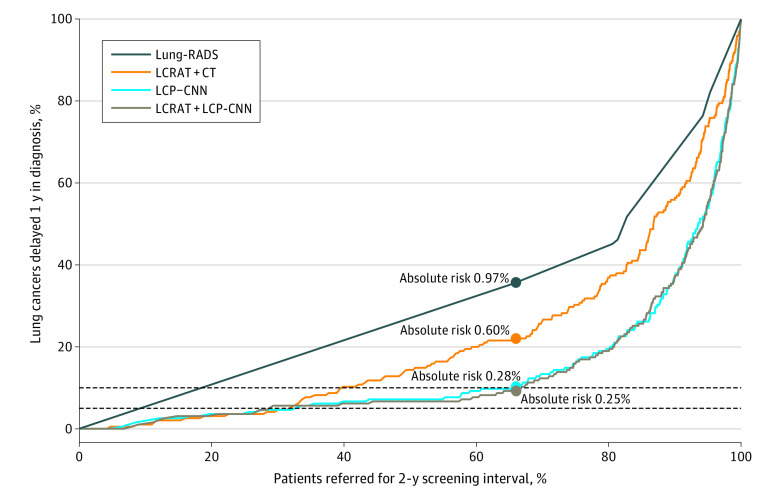
Percentage of Lung Cancers That Would Have Been Delayed 1 Year in Diagnosis vs Percentage of Patients Who Would Have Been Referred for a 2-Year Screening Interval Patients are referred to biennial screening following a nonmalignant abnormal screen result on computed tomography (CT) in the National Lung Screening Trial. The percentage of patients referred for a 2-year screening interval is among those not diagnosed with lung cancer before their next screen. Absolute risks are the absolute risk of lung cancer in 1 year among individuals assigned to biennial screening when 66% of individuals are assigned a 2-year screening interval (note that 66% of individuals have an American College of Radiology recommendation for lung nodules [Lung-RADS], version 1.1, score ≤2). Assigning everyone to biennial screening results in 1-year lung cancer absolute risk of 1.8%. The dotted lines show 5% and 10% of 1-year screen-detected lung cancers being delayed in diagnosis due to assigning biennial screening. LCP-CNN indicates recalibrated Lung Cancer Prediction Convolutional Neural Network; LCRAT + CT, Lung Cancer Risk Assessment Tool plus CT. Results for LCRAT + CT differ from those in Robbins et al^7^ due to the different analysis population.

The LCP-CNN, LCRAT + CT, and LCRAT plus LCP-CNN models perform very similarly in their ability to assign people who were not diagnosed with lung cancer before the next screen to biennial screening until 5% of cancers are delayed in diagnosis (Figure), assigning 33.2% to biennial screening for LCP-CNN, 32.0% for LCRAT + CT, and 29.1% for LCRAT plus LCP-CNN (when 5% of cancers are delayed in diagnosis) (eTable 1 in Supplement 1). These values compare with the 5% of individuals who were not diagnosed with lung cancer before the next screen being assigned biennial screening when 5% of cancers are delayed in diagnosis if individuals were randomly assigned biennial screening. However, above this threshold, LCP-CNN assigns a larger proportion of people to biennial screening compared with LCRAT + CT. For example, if 10% of cancers were delayed in diagnosis, 66.4% and 67.7% of people who were not diagnosed with lung cancer before the next screen would be assigned biennial screening under LCP-CNN and LCRAT plus LCP-CNN, respectively, compared with 40.3% under LCRAT + CT (*P* < .001 for both LCP-CNN vs LCRAT + CT and LCRAT plus LCP-CNN vs LCRAT + CT). In contrast, Lung-RADS at the scoring threshold of 2 or less would have also referred 66.1% of participants for biennial screening (7026 of 10 636) but would have delayed the diagnosis for a much higher percentage of cancers (69 of 195 [35.4%]; *P* < .001 vs 10%) (Table 3). Results by nodule size are reported in the eMethods and eTable 1 in Supplement 1.

**Table 3.** Percentage of Cancers That Would Be Delayed in Diagnosis and Patients Without Cancer Who Would Be Assigned Biennial Screening Under Lung-RADS Score

Lung-RADS score used as threshold^a^	Cancers delayed in diagnosis, No. (%)	Patients without cancer assigned biennial screening, No. (%)	Absolute risk of delaying a cancer in diagnosis among those assigned biennial screening, %
≤4B	195 (100)	10 636 (100)	1.80
≤4A or 4B	160 (82.1)	10 162 (95.6)	1.55
≤4A	149 (76.4)	10 057 (94.6)	1.46
≤3, 4A, or 4B	101 (51.8)	8863 (83.3)	1.13
≤3 or 4A	90 (46.2)	8722 (82.0)	1.02
≤3	88 (45.1)	8639 (81.2)	1.01
≤2	69 (35.4)	7026 (66.1)	0.97

Abbreviation: Lung-RADS, American College of Radiology recommendations for lung nodules, version 1.1.

^a^ Insufficient information was available to assign some individuals to 1 Lung-RADS category, so they were assigned to a grouped category (eg, 3 or 4A).

Characteristics of the cancers with delayed diagnoses when 5% of cancers were delayed in diagnosis under each model (LCRAT + CT, LCP-CNN, and LCRAT plus LCP-CNN) are given in eTable 2 in Supplement 1. Nine of the 10 cancers delayed in diagnosis by LCP-CNN would also be delayed by LCRAT plus LCP-CNN, whereas only 3 would also be delayed by LCRAT + CT. Most of the delayed cancers by each approach had small (4- to 5-mm) nodules at the preceding screen (LCRAT + CT: 6 of 10 [60.0%]; LCP-CNN: 6 of 10 [60.0%]; LCRAT plus LCP-CNN: 5 of 10 [50.0%]). For LCP-CNN and LCRAT plus LCP-CNN, 5 of the 9 cancers with known stage were stage 1 or 2, stages at which delaying the detection by 1 year may substantially reduce the chance of cure (compared with the LCRAT + CT model, in which 3 of the 9 were stage 1 or 2). When 10% of cancers would be delayed in diagnosis, 10 of 18 cancers (55.6%) with a known stage delayed under LCRAT + CT were stage 1 or 2 compared with 14 of 19 (73.7%) under LCP-CNN.

### Screens With a Lung-RADS Score of 2

Of the 10 831 abnormal screen results presumed nonmalignant included in this analysis, 7095 had a Lung-RADS score of 2, with 69 cancers diagnosed as a result of the following screen. When restricted to screens with a Lung-RADS score of 2, LCRAT + CT overestimated the risk of cancer (ratio of expected to observed cases, 1.39; 95% CI, 1.10-1.76), whereas LCP-CNN had excellent calibration (ratio of expected to observed cases, 1.01; 95% CI, 0.79-1.27) (eTable 3 in Supplement 1). The AUCs were slightly lower than when all abnormal presumed nonmalignant screen results were included (LCRAT + CT: 0.75 vs 0.79; LCP-CNN: 0.81 vs 0.87). The absolute risk of delaying a cancer’s diagnosis when 80% of screens with an abnormal presumed nonmalignant nodule are assigned biennial screening was 0.28% under LCP-CNN, half the risk under LCRAT + CT (0.55%; *P* = .09) (eTable 4 in Supplement 1). Among noncases with nodules 13 mm or greater in diameter, 0% and 1.6% were assigned biennial screening under LCRAT + CT and LCP-CNN, respectively.

## Discussion

Our findings are a proof of principle that deep learning algorithms could be used to safely assign many people with small nodules to biennial lung cancer screening with LDCT, thereby potentially reducing its harms. We recalibrated the existing LCP-CNN deep learning algorithm for risk of immediate malignant findings to predict 1-year risk following an abnormal presumed nonmalignant screen result. The recalibrated LCP-CNN had substantially higher AUC (0.87) than LCRAT + CT (0.79) or Lung-RADS (0.69). Incorporating additional risk factors into LCP-CNN via LCRAT did not increase the AUC. The absolute risk of delaying a cancer diagnosis by 1 year, when 66% of abnormal presumed nonmalignant screen results were assigned to biennial screening, was lowest under the recalibrated LCP-CNN (0.28%) compared with LCRAT + CT (0.60%) or Lung-RADS (0.97%). Deep learning algorithms may improve risk stratification by referring people with currently malignant nodules for definitive diagnosis and providing an appropriate screening interval for people with abnormal presumed nonmalignant lung nodules. The ability to assign individuals to biennial screening may make screening feasible for countries with limited resources.

The recalibrated LCP-CNN outperformed LCRAT + CT and Lung-RADS, despite not being optimized to predict 1-year cancer risk, suggesting that deep learning was able to identify CT image features that predict malignant findings that were not included in LCRAT + CT. The fact that the AUC for recalibrated LCP-CNN was not improved by including the LCRAT score (LCRAT plus LCP-CNN) suggests that traditional risk factors such as age and smoking do not provide additional information beyond nodule features accounted for by the LCP-CNN score. Further performance gains might result from a CNN optimized for 1-year risk.

Around half the cancers that would have been delayed in diagnosis by each of these methods were diagnosed at stage 3 or 4, even with yearly screening, despite the goal of screening to detect localized cancers, when there is a high chance of curative treatment. Cancers diagnosed at stage 1A may still be localized if they had been diagnosed 1 year later, though delays in lobectomy of stage 1A squamous cell lung cancers have been shown to reduce survival.^19^ Under the current standard of care, defined by Lung-RADS 2022 in the US or the NHS England Targeted Lung Health Checks Programme protocol in the UK, many screens with large nodules would be referred for diagnostic follow-up; these individuals would only be assigned a 2-year screening interval under LCP-CNN after having a prevalent cancer ruled out. If any reason for concern was identified on a surveillance screen, diagnostic follow-up would continue; once any immediate concern was ruled out, the patients would be assigned to a longer screening interval based on LCP-CNN. Therefore, no one who had cancer diagnosed as a result of follow-up from an abnormal screen result would be assigned a longer screening interval. When only screens with a Lung-RADS score of 2 were considered for biennial screening, the absolute risk of delaying a cancer diagnosis when 80% of screens were assigned biennial screening was 0.28% under LCP-CNN and 0.55% under LCRAT + CT. These harms of delaying a diagnosis must be weighed against the harms of overscreening individuals, which can result in anxiety and, less frequently, invasive follow-up. Additional research on the impact of longer screening intervals on the stage distribution of screen-detected lung cancers and the proportion of cancers diagnosed as interval cancers would be important if biennial screening was implemented for low-risk individuals.

Our findings suggest the potential to individualize the entire lung cancer screening continuum of care by continually updating risks from entry to exit, via integration into electronic health record (EHR) clinical work flow. First, the decision to enter screening could depend on a shared decision-making process based on individual life-years gained from screening, a proxy for benefit-harm tradeoff from screening.^20^ A clinical decision support tool, Decision Precision+,^21^ already allows an EHR system to pull available risk factor information to calculate risks from the LCRAT and life-years gained from screening models.^20^ After a CT screen, if no cancer were detected, the CT image would be in the EHR, annotated by radiologist-determined features, and if a nodule were identified, the LCP-CNN score could be calculated and recorded in the EHR. Next, risk could be updated by the EHR using either LCRAT + CT (following a negative screen result^6^) or recalibrated LCP-CNN (following an abnormal presumed nonmalignant screen result), and a screening interval recommended. As new deep learning and statistical models are validated and imaging technology improves, the new models could be inserted into clinical decision support tools to improve screening interval recommendations. Screening would continue until the estimated life-years gained from screening goes below a minimum threshold deemed worthwhile to continue screening,^20^ at which point anyone with a negative screen result could exit screening.

### Limitations

Our study has some limitations. Although 2 randomized clinical trials suggest that biennial screening is safe,^2,3^ the reduction in screening effectiveness from biennial screening is unknown. Within the NLST, data do not exist to identify whether a cancer developed from any specific nodule; therefore, it is not possible to definitively know specifically which (or whether any) of the nodules evaluated by LCP-CNN progressed to cancer. The LCP-CNN model required the nodule to be identified before it can be applied, and we only evaluate its performance in solid nodules. Currently, the NLST is the only large-scale lung screening study whose images are publicly available, and external validation is important before this approach could be implemented clinically. Although LCP-CNN was developed using some NLST images, which could potentially bias the results toward overestimating its performance, the LCP-CNN has performed well in external validation studies^9,11^; we used only the cross-validated LCP-CNN predictor that left out the image in question, though other images from the same individual could be included; and we used an optimism-adjusted AUC. The LCRAT + CT model was also developed using NLST data; similarly, we used cross-validation and optimism-adjusted AUCs to reduce the potential bias. Although LCP-CNN has been externally validated, the single recalibration parameter for recalibrated LCP-CNN might need to be recalculated for use in populations with substantially different lung cancer risk. The Lung-RADS score was updated in November 2022; while most nodules would be assigned the same Lung-RADS score, we do not have the raw data required to rescore each nodule.

Other limitations reduced the predictive abilities of the models. We had no LCP-CNN score for screens containing only 4-mm nodules (as measured by Optellum Ltd), which have the least risk and are the best candidates for interval extension; the performance of LCP-CNN would likely improve if all nodule sizes were evaluated. We did not have nodule volume to input into LCRAT + CT, which may further improve its performance. Improvements in imaging technology since the NLST mean that the scans evaluated in this study do not represent current practice and may also improve the performance of LCRAT + CT and LCP-CNN.

## Conclusions

Lung cancer screening could demonstrate the first fully individualized risk-benefit–based approach through a lifetime of screenings. Deep learning algorithms could be crucial to prioritize people for workup of suspicious nodules and to lengthen the screening interval for people with low-risk nodules. As programs evaluate the cost-effectiveness and feasibility of delivering interventions that challenge health care capacity, risk-based approaches may inform decisions on whether and how to implement lung cancer screening.

## Notes

Supplement 1. **eMethods.** Classification of Lung Cancers, Models, and Analyses **eTable 1.** Percentage of Individuals Who Were Not Diagnosed With Cancer Who Would Be Safely Assigned Biennial Screening, by the Percentage of Cancers Delayed in Diagnosis **eTable 2.** Details of Incorrectly Ruled-Out Cancers According to the Models When 5% of Cancers Are Delayed in Diagnosis **eTable 3.** Model Performance for Each Model When Restricted to Screens With a Lung-RADS Score of 2 **eTable 4.** The Absolute Risk of Delaying a Cancer Diagnosis, and the Percentage Safely Assigned Biennial Screening, for Each Model When Restricted to Screens With a Lung-RADS Score of 2 **eFigure 1.** CONSORT Flowchart Showing Study Eligibility **eFigure 2.** Distribution of 1-Year Lung Cancer Risk Among People With Nonmalignant Abnormal Screens in the National Lung Screening Trial
eReferences

Supplement 2.
Data Sharing Statement
