# Supplementary material for: Recalibration of a Deep Learning Model for Low-Dose Computed Tomographic Images to Inform Lung Cancer Screening Intervals
Source: JAMA Netw Open. 2023 Mar 16;6(3):e233273. doi: 10.1001/jamanetworkopen.2023.3273 (PMC10020880; doi:10.1001/jamanetworkopen.2023.3273)
Supplement: Supplement 1. — eMethods. Classification of Lung Cancers, Models, and Analyses eTable 1. Percentage of Individuals Who Were Not Diagnosed With Cancer Who Would Be Safely Assigned Biennial Screening, by the Percentage of Cancers Delayed in Diagnosis eTable 2. Details of Incorrectly Ruled-Out Cancers According to the Models When 5% of Cancers Are Delayed in Diagnosis eTable 3. Model Performance for Each Model When Restricted to Screens With a Lung-RADS Score of 2 eTable 4. The Absolute Risk of Delaying a Cancer Diagnosis, and the Percentage Safely Assigned Biennial Screening, for Each Model When Restricted to Screens With a Lung-RADS Score of 2 eFigure 1. CONSORT Flowchart Showing Study Eligibility eFigure 2. Distribution of 1-Year Lung Cancer Risk Among People With Nonmalignant Abnormal Screens in the National Lung Screening Trial eReferences [file jamanetwopen-e233273-s001.pdf]

Supplementary Online Content

Landy R, Wang VL, Baldwin DR, et al. Recalibration of a deep learning model for low-dose computed tomographic images to inform lung cancer screening intervals. *JAMA Netw Open*. 2023;6(3):e233273. doi:10.1001/jamanetworkopen.2023.3273

eMethods. Classification of Lung Cancers, Models, and Analyses
eTable 1. Percentage of Individuals Who Were Not Diagnosed With Cancer Who Would Be Safely Assigned Biennial Screening, by the Percentage of Cancers Delayed in Diagnosis
eTable 2. Details of Incorrectly Ruled-Out Cancers According to the Models When 5% of Cancers Are Delayed in Diagnosis
eTable 3. Model Performance for Each Model When Restricted to Screens With a Lung-RADS Score of 2
eTable 4. The Absolute Risk of Delaying a Cancer Diagnosis, and the Percentage Safely Assigned Biennial Screening, for Each Model When Restricted to Screens With a Lung-RADS Score of 2
eFigure 1. CONSORT Flowchart Showing Study Eligibility
eFigure 2. Distribution of 1-Year Lung Cancer Risk Among People With Nonmalignant Abnormal Screens in the National Lung Screening Trial
eReferences

This supplementary material has been provided by the authors to give readers additional information about their work.

© 2023 Landy R et al. *JAMA Network Open*.

eMethods. Classification of Lung Cancers, Models, and Analyses

Linked-Year Method for Classification of Screen-Detected Lung Cancers

This information is reported in the supplement to Kovalchik et al (1): The classification of screen-detected lung cancers was based on the results of the diagnostic follow-up occurring within one year of a linked screen. A screen link started at the screen (T0, T1, or T2) and extended forwards from the end of the current diagnostic chain to the next event. An event could be another procedure, a lung cancer diagnosis, or another screen. If there was no next event, the next event was a screen, or the next event occurred more than 12 months after the current end of the chain, the chain ended. If the next event was a lung cancer within one year, then that cancer was considered screen-linked. Otherwise the next event was another procedure within one year, and that became the new end of the chain and the process repeated.

A positive screen or report of a lung cancer diagnosis triggered the completion of a Diagnostic Evaluation form, where information on procedures and lung cancer diagnoses were captured. The study attempted to collect medical records for any follow-up a participant sought following a positive screen, for up to one year after the screen, or up to two years for nodules found on T2 screens that were either newly detected or showed growth from previous screens. Follow-up beyond one year (or two years for T2 screens) could be collected if the screening center determined that the follow-up was prompted by the screen. If the trial learned of a lung cancer diagnosis not resulting from an NLST screen (usually from either a participant self-report on the Annual Study Update or from a death certificate), the trial attempted to collect records back to whatever non-trial exam or initial presentation with symptoms led to the diagnosis.

Review of the Lung Cancer Risk Assessment Tool (LCRAT)

For all details and parameter estimates, see (2). Briefly, the LCRAT estimates a person's risk (usually 5-year risk, but in this paper, we calculate 1-year risk to match the 1-year screening intervals in the NLST) based on their demographics (age, race, gender, education), smoking (duration, intensity, and quit-years), and other lung-cancer risk factors (BMI, emphysema, and first-degree family history of lung-cancer). The LCRAT consists of two Cox sub-models, one for risk of lung-cancer and the other for the competing-risk of death. The LCRAT was fit in the PLCO control arm and has been validated in the chest-radiography arms of the PLCO and NLST, as well as the NIH-AARP cohort, and the ACS CPS-II cohort (2).

The LCRAT+CT model

For all details, see (3). Briefly, LCRAT+CT is a discrete-time Markov log-binomial risk model for 1-year risk of lung-cancer (4). LCRAT+CT first calculates 1-year pre-screening risk using the

© 2023 Landy R et al. *JAMA Network Open*.

Lung Cancer Risk Assessment Tool (LCRAT)(2). We recently extended LCRAT+CT to predict next-screen risk after a non-malignant abnormal CT screen(3). LCRAT+CT calculates risk as the LCRAT 1-year pre-screening risk raised to an exponent, where the exponent is calculated as the sum of the regression coefficients corresponding to features of the abnormal CT. The LCRAT+CT was originally fit to 12,993 non-malignant abnormal CT-screens in the NLST, where 235 lung-cancers were detected in 1-year (3), and we use those estimates. As a sensitivity analysis, we re-fit the model to the 10,831 abnormal screens in the NLST that also have an LCP-CNN score (195 lung-cancers detected in 1-year), to ensure comparability of comparisons with LCP-CNN. As with LCP-CNN, for each nodule we used an 8-fold cross-validated LCRAT+CT score that was not fit to that nodule, using the same folds as for LCP-CNN. The re-fitted LCRAT+CT showed good cross-validated internal-calibration (195 cases observed vs. 199.2 predicted, p=0.77) and discrimination (optimism-corrected AUC=0.77).

The Lung Cancer Prediction Convolutional Neural Network (LCP-CNN)

We used an AI developed for discrimination of malignant and benign nodules, to evaluate whether it could improve a clinical model for the prediction of a screening patient's likelihood of developing cancer over the upcoming year. Optellum's LCP-CNN is an AI designed to distinguish benign lung nodules from malignancies using only low-dose CT data, and without any assistance from patient clinical factors such as age or smoking history. They trained their AI using specially curated data from the NLST, and validated it on external (non-screening) sources from several other sites (5, 6). The LCP-CNN computes a score from 0-100 to each nodule it analyses, where the analysed nodule must be indicated in 3D on the CT by a clinician. It is thus incapable of scoring a CT on which a nodule does not appear, such as those which are purely negative findings in the NLST.

We have obtained a subset of the cross-validated NLST LCP-CNN scores described in Massion et al (5) directly from Optellum for this work. While the original data described in Massion et al contains one score per nodule per screening study, this current work requires only one score per screening round, so only the highest-scoring nodule's score was used for any given patient-screen. This dataset only includes patients with nodules listed in the NLST metadata as being 5mm or over, and explicitly excludes nodules classified as being ground glass opacities (GGOs), because those criteria were part of the initial data selection protocol described in Massion et al. It was also incapable of scoring nodules at the very periphery of a CT (without full support for a bounding box of the size fed into the AI), so a small number of CTs were missing scores for that reason. As well as reviewing all CTs on which one or more nodules was recorded, a medical doctor or medical student, under expert supervision from University of Oxford Radiologists, reviewed all CTs of patients recorded as having developed lung cancer, and fully reviewed and extended their mark-up and metadata. Additional nodules not listed in the NLST metadata were also added as long as they were not fully calcified (since fully calcified nodules were not

© 2023 Landy R et al. *JAMA Network Open*.

considered “positive findings” in the original NLST data). Finally, this data represents the eight cross-validation splits described in Massion et al, so is technically a set of scores from eight different AIs, all trained with a different combination of patients on the same task.

Recalibrating the LCP-CNN to predict 1-year lung cancer risk

The LCP-CNN predicts risk of immediate malignancy given nodule image features. We recalibrated the LCP-CNN score to predict 1-year lung cancer risk following a non-malignant abnormal screen by fitting a logistic regression model where the LCP-CNN score is the sole covariate. When multiple nodules on a CT image have an LCP-CNN score, we considered only the nodule with the highest LCP-CNN score. The model is

$$\text{1-year risk} = \frac{\exp(-3.07 + 0.66*\text{logit(LCP-CNN score)})}{1+ \exp(-3.07 + 0.66*\text{logit(LCP-CNN score)})}$$

	coefficient (95% CI)	p-value
Intercept	-3.07 (-3.23, -2.91)	<0.001
logit(LCP-CNN score)	0.66 (0.58, 0.73)	<0.001

The negative intercept reflects on the much lower absolute risks attainable for 1-year prediction versus immediate prediction of malignancy. We use the logit of the LCP-CNN score as the covariate. The OR is exp(0.66)=1.93 per unit logit increase in the LCP-CNN score ($p<<0.0001$). Thus, although the LCP-CNN score is meant to predict immediate malignancy, it is also strongly predictive of 1-year lung-cancer risk for those with a non-malignant abnormal screen.

We used 8-fold cross-validation to check the calibration of the model, using the same folds as LCP-CNN was developed using, i.e. we fit the model to 7/8 of the data to make a prediction for the remaining 1/8. In this way, no observation contributed to the model fit used to make a prediction for it. Of the 195 lung-cancers, the model predicted 195.62 ($p>0.9$), indicating good calibration for predicting 1-year lung-cancer risk. The recalibrated LCP-CNN score had optimism-corrected AUC=0.87.

© 2023 Landy R et al. *JAMA Network Open*.

Combining LCRAT and the LCP-CNN score: The LCRAT+LCPCNN model

We combined the LCRAT and LCP-CNN scores by including them as covariates (on the logit scale) in a logistic regression model for 1-year lung-cancer following a non-malignant abnormal screen. When multiple nodules on a CT image have an LCP-CNN score, we considered only the nodule with the highest LCP-CNN score. The model is:

	coefficient (95% CI)	P-value
Intercept	-0.95 (-2.07, 0.17)	0.098
Logit (LCRAT 1-year risk)	0.39 (0.18, 0.60)	<0.001
Logit (LCP-CNN score)	0.64 (0.57, 0.72)	<0.001

The LCRAT score has OR of exp(0.39)=1.5 per unit logit increase in the LCRAT risk. The increased OR for the LCRAT score reflects on the fact that having a non-malignant abnormal screen increases subsequent risk of cancer. Including the LCRAT covariate improves the model fit versus only using the LCP-CNN score (p=0.001).

We used 8-fold cross-validation to check the calibration of the model, using the same folds as LCP-CNN was developed using, i.e. we fit the model to 7/8 of the data to make a prediction for the remaining 1/8. In this way, no observation contributed to the model fit used to make a prediction for it. Of the 195 lung-cancers, the model predicted 195.62 (p>0.9), indicating good calibration for predicting 1-year lung-cancer risk.

The LCRAT+LCPCNN score had optimism-corrected AUC=0.87, very similar to the recalibrated LCP-CNN. Thus, although including LCRAT improves the model, the LCP-CNN score suffices to powerfully predict 1-year lung-cancer risk. This finding suggests that some nodules are premalignant and become malignant in 1 year. Thus features of current (premalignant) nodules remain relevant to predicting malignancy risk in 1-year.

Sample size

In total, there were 13,654 abnormal LDCTs in the first and second screening rounds of the NLST that did not result in a lung cancer diagnosis (7). Of these, 12,993 were used to develop the LCRAT+CT (3), as anyone who did not have a screen at the following screening round was excluded (N=661). Of these, 10,831 were identified as having a nodule with maximum diameter ≥5mm by Optellum, and therefore have an LCP-CNN score, and are included in our analysis dataset. We note that in the NLST meta-data, the largest nodule on 12% of these screens had a mean diameter of 4mm.

Results by nodule size

When 5% of cancers are delayed in diagnosis, LCRAT+CT can assign more small nodules (4-5mm as reported in the NLST metadata) to biennial screening (56.1% vs 47.7% for LCP-CNN,

© 2023 Landy R et al. *JAMA Network Open*.

p<0.001), whereas LCP-CNN assigned more larger nodules (≥8mm) to biennial screening (15.7% vs 3.6%, p<0.001). When 10% of cancers are delayed in diagnosis, LCP-CNN's increased ability to assign biennial screening is especially notable for large nodules (≥11mm diameter) compared to LCRAT+CT (27.1% vs 1.8%, p<0.001). Results in which thresholds of 20% and 35% of cancers are delayed in diagnosis are also shown in **Table S1**.

Sensitivity analysis: Restricted to one screen per person

When the sample was restricted to one screen per person, a total of 7,495 screens were included in the analysis (3,704 from T0 and 3,791 from T1), with 158 screen-detected lung cancers. The AUC for the recalibrated LCP-CNN score (0.88) was greater than that of LCRAT+CT (0.79, p<0.001) and LungRADS (0.71, p<0.001), and did not differ from the combined LCRAT+LCPCNN (0.88, p=0.1449).

Lung-RADS would assign 64% of abnormal presumed non-malignant screens to biennial screening at a threshold of ≤2. When 64% of abnormal presumed non-malignant screens were assigned biennial screening under LCRAT+CT and LCP-CNN, the absolute risk of delaying a cancer diagnosis was 0.98% under Lung-RADS, 0.65% under LCRAT+CT (p=0.09 vs. Lung-RADS), and 0.25% under recalibrated LCP-CNN (p<0.001 vs. Lung-RADS and p=0.005 vs. LCRAT+CT).

© 2023 Landy R et al. *JAMA Network Open*.

eTable 1. Percentage of Individuals Who Were Not Diagnosed With Cancer Who Would Be Safely Assigned Biennial Screening, by the Percentage of Cancers Delayed in Diagnosis

	**% safely assigned biennial screening, by % cancers delayed in diagnosis:**			
**Model**	**5% delayed**	**10% delayed**	**20% delayed**	**35% delayed**
**All nodules**				
LCRAT+CT	32.0%	40.3%	59.9%	79.6%
LCP-CNN	33.2%	66.4%	81.3%	90.1%
LCRAT+LCPCNN	29.1%	67.7%	81.7%	90.1%
**4-5 mm**				
LCRAT+CT	56.1%	67.1%	87.1%	97.8%
LCP-CNN	47.7%	84.8%	94.8%	98.6%
LCRAT+LCPCNN	42.1%	86.3%	94.9%	98.5%
**6-7 mm**				
LCRAT+CT	34.6%	45.5%	70.0%	91.0%
LCP-CNN	35.0%	71.5%	87.4%	95.0%
LCRAT+LCPCNN	31.0%	72.6%	87.6%	94.9%
**8-10 mm**				
LCRAT+CT	5.7%	10.7%	31.3%	62.7%
LCP-CNN	21.7%	53.2%	72.8%	86.6%
LCRAT+LCPCNN	18.8%	54.5%	73.6%	86.9%
**11-13 mm**				
LCRAT+CT	0.9%	1.2%	6.5%	27.3%
LCP-CNN	10.4%	30.0%	52.7%	69.9%
LCRAT+LCPCNN	7.1%	32.7%	55.0%	70.6%
**14+ mm**				
LCRAT+CT	0.9%	2.2%	9.6%	35.0%
LCP-CNN	6.2%	25.1%	40.8%	57.9%
LCRAT+LCPCNN	5.2%	25.2%	41.1%	58.0%

© 2023 Landy R et al. *JAMA Network Open*.

eTable 2. Details of Incorrectly Ruled-Out Cancers According to the Models When 5% of Cancers Are Delayed in Diagnosis

		Calculated Risk by Model			Patient Characteristics				Nodule Characteristics			Histological type	Stage at diagnosis
	Case number	LCRAT + CT	LCP-CNN	LCRAT+ LCPCNN	Quit-years	BMI category	Pack-years	Emphysema	Mean nodule diameter (mm, NLST metadata)*	Location(s)	N nodules		
LCRAT + CT	1	0.002	0.055	0.034	1-5	Obese	50+	No	No GG, 4-5	Upper lobe	1	Adenocarcinoma	1A
	2	0.003	0.026	0.020	6-10	Overweight	40-50	Yes	No GG, 4-5	Lower lobe	1	Squamous cell carcinoma	1A
	3	0.003	0.034	0.024	11+	Obese	50+	No	No GG, 6-7	Lower lobe	1	Adenocarcinoma	1B
	4	0.004	0.015	0.012	Current smoker	Obese	40-50	No	No GG, 6-7	Right middle lobe, Upper lobe, Lower lobe, Lingula	4	Adenocarcinoma	3A
	5	0.005	0.017	0.016	11+	Obese	50+	Yes	No GG, 4-5	Upper lobe	2	Neuroendocrine carcinoma, NOS	3B
	6	0.002	0.002	0.002	11+	Obese	50+	Yes	No GG, 4-5	Right middle lobe	1	Small cell carcinoma	4
	7	0.004	0.001	0.001	6-10	Overweight	30-40	Yes	No GG, 6-7	Lower lobe	1	Non-small cell carcinoma	4
	8	0.005	0.02	0.014	1-5	Overweight	40-50	No	No GG, 6-7	Lower lobe	1	Adenocarcinoma	4
	9	0.002	0.036	0.028	6-10	Overweight	50+	Yes	No GG, 4-5	Lower lobe	1	Small cell carcinoma	4
	10	0.006	0.002	0.002	Current smoker	Overweight	30-40	Yes	No GG, 4-5	Lower lobe	1	Adenocarcinoma	unknown
LCP-CNN	11	0.006	<0.001	<0.001	1-5	Overweight	50+	Yes	No GG, 4-5	Upper lobe	1	Squamous cell carcinoma	1A
	12	0.011	0.001	0.001	1-5	Overweight	50+	No	GG, <20	Lower lobe	1	Adenocarcinoma	1A
	13	0.035	0.002	0.002	1-5	Obese	50+	No	No GG, 8-10	Upper lobe, Lower lobe	3	Adenocarcinoma	1B
	14	0.012	<0.001	<0.001	Current smoker	Overweight	50+	Yes	No GG, 4-5	Upper lobe	1	Squamous cell carcinoma	2A

© 2023 Landy R et al. *JAMA Network Open*.

	15	0.007	0.001	0.001	Current smoker	Normal	40-50	Yes	No GG, 4-5	Lower lobe	1	Small cell carcinoma	2B
	16	0.012	<0.001	0.001	Current smoker	Normal	50+	Yes	No GG, 6-7	Upper lobe	1	Small cell carcinoma	3A
	6	0.002	0.002	0.002	11+	Obese	50+	Yes	No GG, 4-5	Right middle lobe	1	Small cell carcinoma	4
	7	0.004	0.001	0.001	6-10	Overweight	30-40	Yes	No GG, 6-7	Lower lobe	1	Non-small cell carcinoma	4
	17	0.009	0.001	0.001	Current smoker	Overweight	50+	No	No GG, 4-5	Upper lobe	1	Combined small cell carcinoma	4
	10	0.006	0.002	0.002	Current smoker	Overweight	30-40	Yes	No GG, 4-5	Lower lobe	1	Adenocarcinoma	Unknown
LCRAT +LCPC NN	11	0.006	<0.001	<0.001	1-5	Overweight	50+	Yes	No GG, 4-5	Upper lobe	1	Squamous cell carcinoma	1A
	12	0.011	0.001	0.001	1-5	Overweight	50+	No	GG, <20	Lower lobe	1	Adenocarcinoma	1A
	13	0.035	0.002	0.002	1-5	Obese	50+	No	No GG, 8-10	Upper lobe, Lower lobe	3	Adenocarcinoma	1B
	14	0.012	<0.001	<0.001	Current smoker	Overweight	50+	Yes	No GG, 4-5	Upper lobe	1	Squamous cell carcinoma	2A
	15	0.007	0.001	0.001	Current smoker	Normal	40-50	Yes	No GG, 4-5	Lower lobe	1	Small cell carcinoma	2B
	16	0.012	<0.001	0.001	Current smoker	Normal	50+	Yes	No GG, 6-7	Upper lobe	1	Small cell carcinoma	3A
	7	0.004	0.001	0.001	6-10	Overweight	30-40	Yes	No GG, 6-7	Lower lobe	1	Non-small cell carcinoma	4
	17	0.009	0.001	0.001	Current smoker	Overweight	50+	No	No GG, 4-5	Upper lobe	1	Combined small cell carcinoma	4
	18	0.028	0.002	0.002	11+	Overweight	30-40	Yes	GG, ≥20	Upper lobe	2	Carcinoma, NOS	4
	10	0.006	0.002	0.002	Current smoker	Overweight	30-40	Yes	No GG, 4-5	Lower lobe	1	Adenocarcinoma	Unknown

*GG: Ground glass nodule

© 2023 Landy R et al. *JAMA Network Open*.

eTable 3. Model Performance for Each Model When Restricted to Screens With a Lung-RADS Score of 2

Model	N screens	Median (Q1, Q3) risk under the specified model (%)	AUC*	Expected number of cancers	Observed number of cancers	Calibration (E/O)
**All nodules**						
LCRAT+CT	7095	0.7 (0.4, 1.6)	75.4%	96	69	1.39 (1.10, 1.76)
LCP-CNN	7095	0.4 (0.2, 1.0)	81.1%	69	69	1.01 (0.79, 1.27)
LCRAT+LCPCNN	7095	0.4 (0.1, 1.0)	81.8%	69	69	1.00 (0.79, 1.27)
**4-5 mm**						
LCRAT+CT	3590	0.5 (0.3, 0.8)	65.4%	24	17	1.39 (0.87, 2.24)
LCP-CNN	3590	0.3 (0.1, 0.6)	68.7%	19	17	1.14 (0.71, 1.83)
LCRAT+LCPCNN	3590	0.3 (0.1, 0.6)	71.8%	19	17	1.11 (0.69, 1.79)
**6-7 mm**						
LCRAT+CT	1972	0.8 (0.4, 1.4)	72.9%	22	19	1.14 (0.72, 1.78)
LCP-CNN	1972	0.5 (0.2, 0.9)	77.5%	16	19	0.82 (0.53, 1.29)
LCRAT+LCPCNN	1972	0.4 (0.1, 0.9)	77.2%	16	19	0.84 (0.54, 1.32)
**8-12mm**						
LCRAT+CT	1118	2.2 (1.3, 3.8)	65.5%	33	16	2.07 (1.27, 3.37)
LCP-CNN	1118	0.9 (0.3, 2.1)	81.8%	19	16	1.18 (0.72, 1.92)
LCRAT+LCPCNN	1118	0.9 (0.3, 2.1)	80.7%	19	16	1.18 (0.72, 1.93)
**13+mm**						
LCRAT+CT	384	3.4 (2.0, 5.3)	66.4%	17	17	0.99 (0.61, 1.59)
LCP-CNN	384	2.3 (0.8, 4.9)	82.4%	15	17	0.87 (0.54, 1.39)
LCRAT+LCPCNN	384	2.3 (0.9, 4.7)	83.5%	15	17	0.87 (0.54, 1.39)

*AUC is optimism-corrected for LCP-CNN and LCRAT+LCPCNN, to reflect that LCP-CNN was re-fit to this data

We note that in LungRADS v1.1, the threshold of <6mm for a score of 2 refers to the average diameter, and the values in this table refer to the maximum diameter. Additionally, the 6mm threshold is only for solid and part solid nodules (i.e. not ground glass nodules), and does not apply to nodules observed on a previous screen which have not grown.

© 2023 Landy R et al. *JAMA Network Open*.

eTable 4. The Absolute Risk of Delaying a Cancer Diagnosis, and the Percentage Safely Assigned Biennial Screening, for Each Model When Restricted to Screens With a Lung-RADS Score of 2

Model	N screens	Observed number of cancers	Absolute risk of delaying a cancer in diagnosis, by % of individuals assigned biennial screening (N cancers delayed in diagnosis/N individuals assigned biennial screening)			% safely assigned biennial screening, by % cancers delayed in diagnosis (N safely assigned biennial screening)			
			**66%**	**80%**	**90%**	**5%**	**10%**	**20%**	**35%**
**All nodules**									
LCRAT+CT	7095	69	0.41% (19/4683)	0.55% (31/5676)	0.64% (41/6386)	15.1% (1063)	41.5% (2917)	53.2% (3737)	71.9% (5051)
LCP-CNN	7095	69	0.26% (12/4683)	0.28% (18/5676)	0.42% (27/6386)	9.5% (667)	21.5% (1514)	75.0% (5270)	86.6% (6082)
LCRAT+LCPCNN	7095	69	0.23% (11/4683)	0.28% (16/5676)	0.44% (28/6386)	13.3% (932)	26.6% (1867)	74.1% (5204)	86.5% (6079)
**4-5 mm**									
LCRAT+CT	3590	17	0.39% (12/3081)	0.38% (13/3433)	0.45% (16/3557)	24.3% (867)	60.0% (2145)	73.6% (2629)	90.4% (3231)
LCP-CNN	3590	17	0.25% (7/2808)	0.28% (9/3247)	0.34% (12/3480)	12.0% (430)	28.0% (999)	86.1% (3077)	94.7% (3384)
LCRAT+LCPCNN	3590	17	0.21% (6/2828)	0.22% (7/3241)	0.37% (13/3467)	17.4% (621)	34.2% (1222)	85.5% (3055)	94.5% (3376)
**6-7 mm**									
LCRAT+CT	1972	19	0.45% (6/1334)	0.77% (13/1681)	0.96% (18/1882)	9.8% (191)	37.1% (724)	50.6% (989)	75.0% (1465)
LCP-CNN	1972	19	0.30% (4/1319)	0.37% (6/1619)	0.60% (11/1826)	9.8% (192)	20.7% (404)	76.8% (1499)	89.2% (1742)
LCRAT+LCPCNN	1972	19	0.31% (4/1305)	0.37% (6/1623)	0.55% (10/1822)	12.6% (246)	25.2% (492)	75.6% (1477)	88.9% (1737)
**8-12mm**									
LCRAT+CT	1118	16	0.43% (1/231)	0.86% (4/466)	0.67% (5/746)	0.5% (5)	4.4% (48)	9.4% (104)	27.7% (305)
LCP-CNN	1118	16	0.21% (1/470)	0.45% (3/667)	0.46% (4/867)	4.1% (45)	9.4% (104)	52.4% (577)	70.3% (775)
LCRAT+LCPCNN	1118	16	0.22% (1/461)	0.45% (3/670)	0.57% (5/879)	5.7% (63)	12.5% (138)	50.6% (558)	71.1% (784)
**13+mm**									
LCRAT+CT	384	17	0.00% (0/34)	1.19% (1/84)	1.12% (2/178)	0.0% (0)	0.0% (0)	3.5% (13)	11.7% (43)
LCP-CNN	384	17	0.00% (0/77)	0.00% (0/127)	0.00% (0/193)	0.0% (0)	1.6% (6)	28.3% (104)	44.7% (164)
LCRAT+LCPCNN	384	17	0.00% (0/79)	0.00% (0/126)	0.00% (0/197)	0.3% (1)	3.5% (13)	27.2% (100)	45.0% (165)

We note that in LungRADS v1.1, the threshold of <6mm for a score of 2 refers to the average diameter, and the values in this table refer to the maximum diameter. Additionally, the 6mm threshold is only for solid and part solid nodules (i.e. not ground glass nodules), and does not apply to nodules observed on a previous screen which have not grown.

© 2023 Landy R et al. *JAMA Network Open*.

eFigure 1. CONSORT Flowchart Showing Study Eligibility

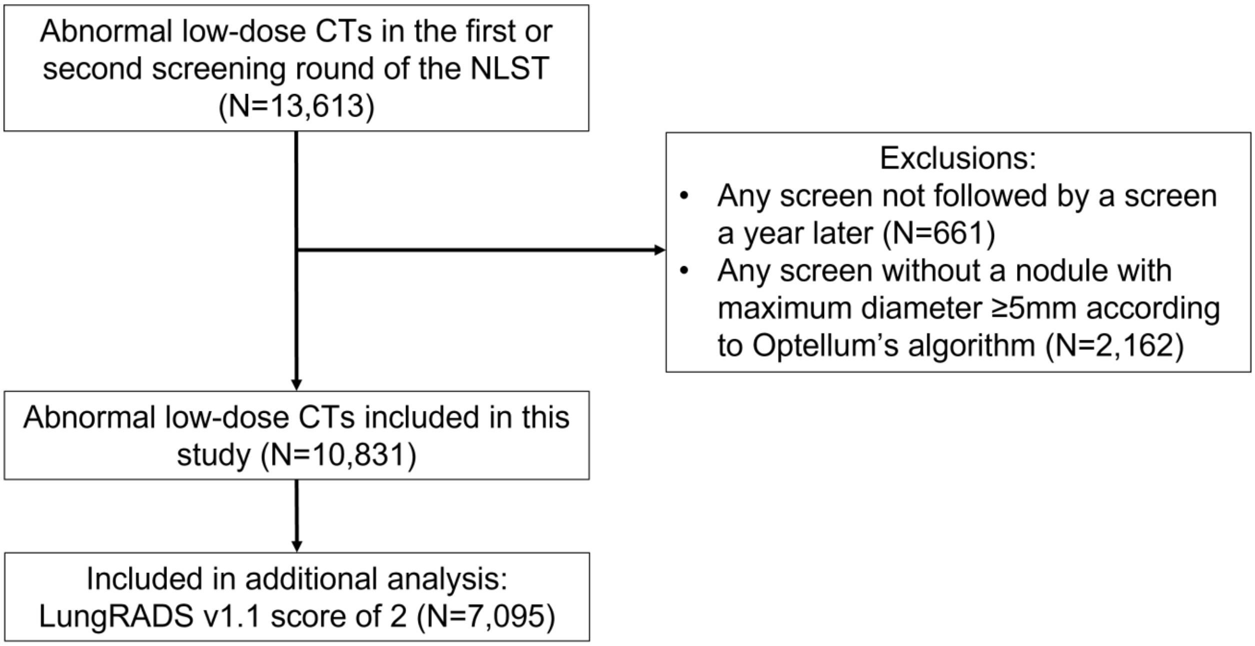

© 2023 Landy R et al. *JAMA Network Open*.

eFigure 2. Distribution of 1-Year Lung Cancer Risk Among People With Nonmalignant Abnormal Screens in the National Lung Screening Trial

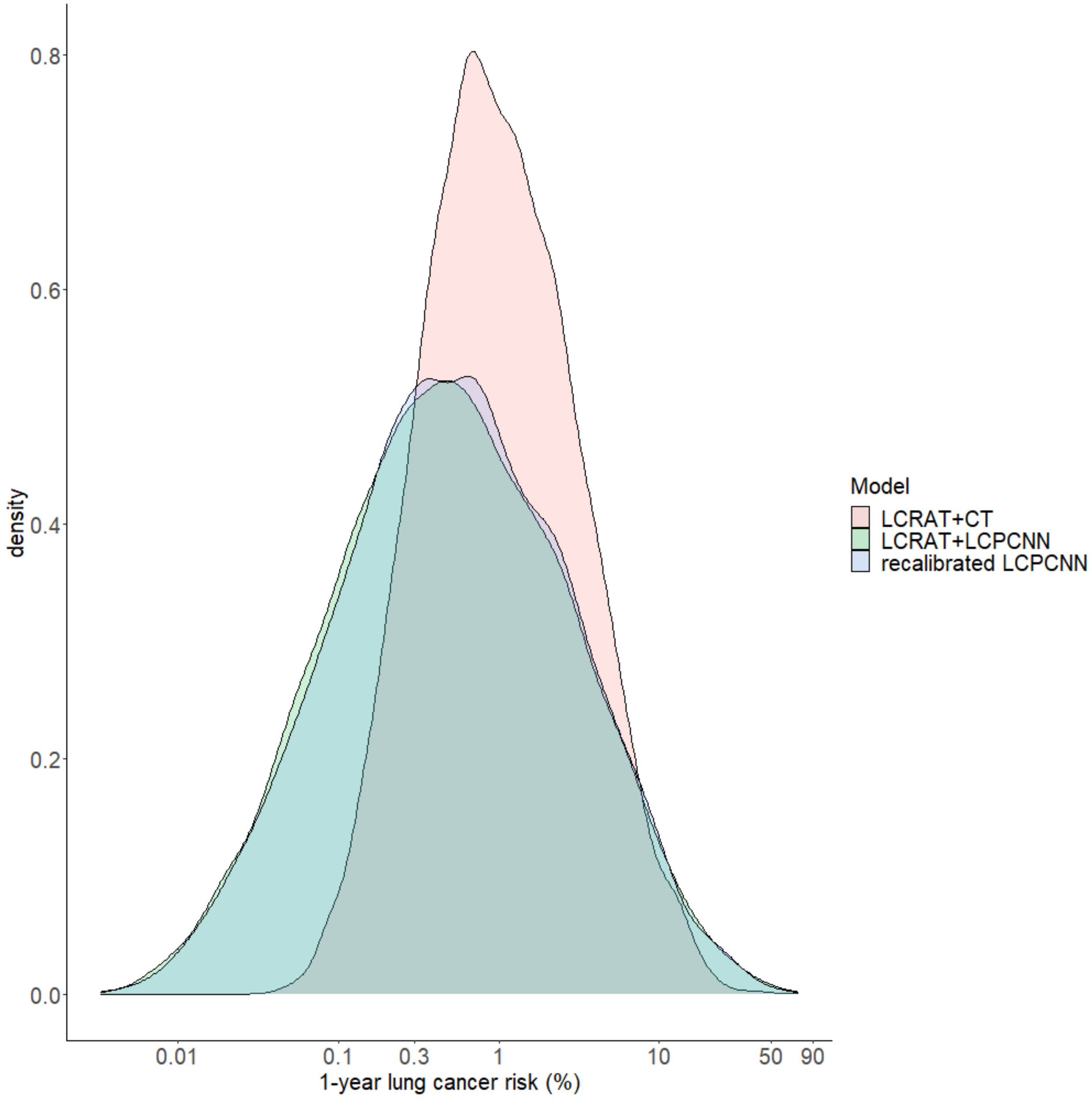

Footnote: The X-axis is plotted on a logit scale to display the very low risks estimable by the recalibrated LCP-CNN and LCRAT+LCPCNN models. Risk distributions for the recalibrated LCP-CNN and the LCRAT+LCPCNN are extremely similar. The mean risk for all 3 models is 1.8%.

© 2023 Landy R et al. *JAMA Network Open*.

eReferences

1. Kovalchik SA, Tammemagi M, Berg CD, Caporaso NE, Riley TL, Korch M, et al. Targeting of low-dose CT screening according to the risk of lung-cancer death. N Engl J Med. 2013;369(3):245-54.
2. Katki HA, Kovalchik SA, Berg CD, Cheung LC, Chaturvedi AK. Development and validation of risk models to select ever-smokers for CT lung cancer screening. JAMA. 2016;315(21):2300-11.
3. Robbins H, Cheung L, Chaturvedi A, Baldwin D, Berg C, Katki H. Management of lung cancer screening results based on individual prediction of current and future lung cancer risk. Submitted.
4. Robbins HA, Berg CD, Cheung LC, Chaturvedi AK, Katki HA. Identification of candidates for longer lung cancer screening intervals following a negative low-dose computed tomography result. J Natl Cancer Inst. 2019;111(9):996-9.
5. Massion PP, Antic S, Ather S, Arteta C, Brabec J, Chen H, et al. Assessing the accuracy of a deep learning method to risk stratify indeterminate pulmonary nodules. American journal of respiratory and critical care medicine. 2020;202(2):241-9.
6. Baldwin DR, Gustafson J, Pickup L, Arteta C, Novotny P, Declerck J, et al. External validation of a convolutional neural network artificial intelligence tool to predict malignancy in pulmonary nodules. Thorax. 2020;75(4):306-12.
7. National Lung Screening Trial Research Team. Reduced lung-cancer mortality with low-dose computed tomographic screening. New England Journal of Medicine. 2011;365(5):395-409.

© 2023 Landy R et al. *JAMA Network Open*.
