# Supplementary material for: Recalibration of a Deep Learning Model for Low-Dose Computed Tomographic Images to Inform Lung Cancer Screening Intervals
Source: JAMA Netw Open. 2023 Mar 16;6(3):e233273. doi: 10.1001/jamanetworkopen.2023.3273 (PMC10020880; doi:10.1001/jamanetworkopen.2023.3273)
Supplement: Supplement 2. — Data Sharing Statement [file jamanetwopen-e233273-s002.pdf]

Data Sharing Statement

Landy. Recalibration of a Deep Learning Model for Low-Dose Computed Tomographic Images to Inform Lung Cancer Screening Intervals. *JAMA Netw Open*. Published March 16, 2023. doi:10.1001/jamanetworkopen.2023.3273

Data
Data available: No

Additional Information
Explanation for why data not available: The LCPCNN score was generated by Optellum Ltd, UK, and we do not have permission to make this data available.
